# Blending of Periodical and Continued Fevers

**Published:** 1856-08

**Authors:** O. C. Gibbs

**Affiliations:** Frewsburg, Chautauque Co.


### ART. IV.—"*Blending of Periodical and Continued Fevers.*"

By O. C. Gibbs, M. D., Frewsburg, Chautauque Co.

In the June number of your Journal, Dr. Flint furnishes a case of fever of disputed type, reported by Dr. W. J. Chenoweth, of Decatur, Illinois, with additional remarks by himself, on the blending of periodical and continued fevers. We propose to add a few remarks, partially confirmatory and partially dissenting from the opinions advanced by Dr. Flint. We quote his statement of the question. He says: "The poison improperly called *marsh miasma* is capable of producing the species of fever called intermitting and remitting, and these only; while the special cause of typhoid fever, be it the matter of contagion or not, will give rise to the species of fever last named, and none other. Now the question is, May these two different morbific agencies act in conjunction within the organism, the processes peculiar to each going on simultaneously, and, consequently giving rise to an union of the symptomatic phenomena peculiar to each?" Dr. Flint is disposed to answer this question in the affirmative, and says: "The affirmative answer to this inquiry by no means involves a pathological absurdity. The old doctrine that two diseases cannot coëxist in the same place and time within the body, is now obsolete. Observation has abundantly established that two essentially distinct fevers may run their respective courses simultaneously. Scarlet fever has been repeatedly known to be thus associated with measles and with small-pox. The supposition, then, that periodical and continued fevers are capable of being blended, is sustained by analogy."

We believe, with Dr. Flint, that miasmatic and typhoid fevers are capable of being blended in the same case. Such cases we are confident we have repeatedly seen. It has been our privilege formerly, to do business in a township in the northern part of which periodical fevers were quite frequent, while in the south part of the same town they were seldom or never known, unless contracted elsewhere. We have once known typhoid fever to prevail within the immediate sphere of our observation. In the south part of the town the cases were distinctly typhoid, while in the north part they were frequently hybrid, presenting the symptoms, variously combined, of periodical and continued fevers.

While agreeing with Dr. Flint in the opinion as above expressed, we are compelled to dissent from the idea advanced in the following extracts:

"I am," he says, "disposed to advance a step beyond an affirmative answer to this question, and to ask whether it be not a rational view of remitting fever to regard it as always involving a blending of the intermittent and the continued. Why do we have in malarious districts, in certain instances, remitting instead of intermitting fever?" * * * * * "The specific effects of the malarious poison are intermittent febrile paroxysms, more or less complete and intense, recurring at regular intervals. In remitting fever an additional pathological element appears to be involved—two separate affections, viz., a continued and a paroxysmal fever, are united. The disease is a hybrid."

The idea, as above expressed, seems very plausible, and coming from such high authority and so close an observer as is Dr. Flint, we are reluctant to express our dissent; yet the hybrid affection, supposed to be formed by a union of the two causes which produce respectively malarious and typhoid fevers, as it has fallen under our observation, is altogether a *different* disease from remittent fever. In most instances remittent fever will bear bloodletting well, and can be converted into an intermittent form within twenty-four hours, and within twenty-four hours more the paroxysms be arrested. Not so with that hybrid affection under consideration; bloodletting in such cases is illy borne, the bowels are unusually sensitive to cathartics, and the febrile affection will continue, notwithstanding the administration of those remedies usually efficacious in the intermittent and remittent forms of fever.

We have never seen reasons for believing that remitting fever was produced by causes distinct and independent from those operative in the production of the intermitting form of fever. Certain it is that, in our hands, the remitting has been promptly convertible into the intermitting, and as promptly arrested. The mongrel affection under consideration, we have never been able to convert at once into a distinct intermittent; it only assumes that form, and frequently not then, after a subsidence of the symptoms indicative of its typhoid complications. But, what is more significant perhaps, the early administration of quinine will usually put an end to its remitting features, and convert it, *not into an intermittent, but into a more distinctly formed continued fever.*

We have thus briefly called attention to two points of Dr. Flint's paper, hoping that the interest of the subject may induce some of our western brethren, who have had more experience with the diversified forms of malarious manifestations, will report the results of their observations.

